# The Mitochondrial Deubiquitinase USP30 Regulates AKT/mTOR Signaling

**DOI:** 10.3389/fphar.2022.816551

**Published:** 2022-02-17

**Authors:** Ruohan Zhang, Serra Ozgen, Hongke Luo, Judith Krigman, Yutong Zhao, Gang Xin, Nuo Sun

**Affiliations:** ^1^ Department of Physiology and Cell Biology, The Ohio State University Wexner Medical Center, Columbus, OH, United States; ^2^ Division of Pharmaceutics and Pharmacology, The Ohio State University College of Pharmacy, Columbus, OH, United States; ^3^ Pelotonia Institute for Immuno-Oncology, The Ohio State University Comprehensive Cancer Center, Columbus, OH, United States

**Keywords:** USP30, parkin, mitophagy, akt, mTOR, leukemia, cancer

## Abstract

Mitophagy is an intracellular mechanism to maintain mitochondrial health by removing dysfunctional mitochondria. The E3 ligase Parkin ubiquitinates the membrane proteins on targeted mitochondria to initiate mitophagy, whereas USP30 antagonizes Parkin-dependent mitophagy by removing ubiquitin from Parkin substrates. The AKT/mTOR signaling is a master regulator of cell proliferation, differentiation, apoptosis, and autophagy. Although mounting evidence suggests that perturbations in the AKT/mTOR signaling pathway may contribute to mitophagy regulation, the specific mechanisms between Parkin/USP30 and AKT/mTOR signaling have not been elucidated. In this study, we employ a set of genetic reagents to investigate the role of Parkin and USP30 in regulating the AKT/mTOR signaling during mitophagy. We demonstrated that, in the setting of mitochondrial stress, the AKT/mTOR signaling is regulated, at least in part, by the activity of Parkin and USP30. Parkin inhibits AKT/mTOR signaling following an *in vitro* mitochondrial stress, thereby promoting apoptosis. However, USP30 overexpression antagonizes the activity of Parkin to sustain AKT/mTOR activity and inhibit apoptosis. These findings provide new insights into Parkin and USP30’s role in apoptosis and suggest that inhibiting USP30 might provide a specific strategy to synergize with AKT/mTOR inhibitors in cancer treatment.

## Introduction

Mitophagy, also known as mitochondria-specific autophagy, is an evolutionarily conserved cellular mechanism to recycle specific mitochondria, by being encapsulated by the structurally double-membrane autophagosome (Youle and Narendra, 2011; Dikic and Elazar, 2018; Saha et al., 2018; Khandia et al., 2019). Mitophagy plays an essential role in maintaining mitochondrial health and metabolic reactions during cellular stress, such as hypoxia or starvation (Palikaras et al., 2018; Killackey et al., 2020).

Mitophagy is highly regulated (Chu, 2011). The PTEN (Phosphatase and tensin homolog)-induced kinase 1 (PINK1) and the cytosolic E3 ubiquitin ligase Parkin are important mitophagy promoters (Narendra et al., 2010; Ding and Yin, 2012; Ge et al., 2020). When mitochondria are damaged, PINK1 accumulates on the outer mitochondrial membrane (OMM) and recruits Parkin from the cytosol to ubiquitinate mitochondrial membrane proteins, including the translocase of the outer membrane subunit 20 (TOM20) (Narendra et al., 2010; Yoshii et al., 2011; Lazarou et al., 2015; Bingol and Sheng, 2016; Palikaras et al., 2018; Killackey et al., 2020). Ubiquitin chains on the mitochondrial membrane tag the mitochondria and induce the organelle’s engulfment by the autophagosome (Narendra et al., 2010; Ding and Yin, 2012; Lazarou et al., 2015; Bingol and Sheng, 2016; Palikaras et al., 2018). Ubiquitin carboxyl-terminal hydrolase 30 (USP30) works as an essential checkpoint for mitophagy initiation (Liang et al., 2015; Kluge et al., 2018; Luo et al., 2021). USP30 is an OMM deubiquitinase that cleaves the Parkin-mediated ubiquitin chains to inhibit mitophagy (Liang et al., 2015; Bingol and Sheng, 2016). Expressing Parkin or inhibiting USP30 can promote mitophagy, improve mitochondrial functions, and rescue the symptoms of certain mitochondrial-related diseases (Bingol et al., 2014; Krishnmurthy and Shradgan, 2014). However, it remains possible that cells may become more vulnerable to specific mitochondrial stresses, and cell death may occur if the mitophagy response is too exuberant (Carroll et al., 2014; Liang et al., 2015; Wanderoy et al., 2020).

Another significant mitophagy regulator is the AKT (Protein kinase B)/mTOR (The mechanistic target of rapamycin) signaling (Kim and Guan, 2015; Soutar et al., 2018; de la Cruz López et al., 2019). The AKT/mTOR signaling is an intracellular pathway that plays a vital role in regulating cell survival (Manning and Toker, 2017; Xu et al., 2020). Previous studies indicate that the AKT/mTOR signaling inhibits mitophagy and promotes cell survival under mitochondrial stress (Akundi et al., 2012; Yang et al., 2017; Soutar et al., 2018; de la Cruz López et al., 2019; Wanderoy et al., 2020). However, it remains unclear how Parkin/USP30 and the AKT/mTOR signaling interact. Of note, during mitochondrial stress, Parkin expression or USP30 inhibition may induce cell apoptosis (Carroll et al., 2014; Liang et al., 2015). Interestingly, the AKT/mTOR pathway is dysregulated and hyperactive in 50–80% of human leukemia cases (Park et al., 2010; Nepstad et al., 2020). AKT hyperactivation correlates with aggressive cancer progression and resistance to a plethora of chemotherapeutics (Arafeh and Samuels, 2019). While targeting The AKT/mTOR pathway could serve as promising strategies for cancer treatment, the efficacy of monotherapy with AKT inhibitors is limited (Fransecky et al., 2015; Estruch et al., 2021). Further investigation of USP30’s function in regulating AKT/mTOR signaling may offer new therapeutic approaches in cancer treatment.

In our study, Hela cells engineered to express Parkin (Hela Parkin cells) were exposed to a cocktail of mitochondrial inhibitors (antimycin plus oligomycin; AO). Consistent with previous observations, the addition of AO triggered rapid PINK1/Parkin mediated-mitophagy *in vitro* (Narendra et al., 2010; Vives-Bauza et al., 2010; Lazarou et al., 2015; Ordureau et al., 2018). In addition, we observed Parkin-dependent AKT downregulation and increased cell apoptosis after AO treatment. In this context, the increased expression of USP30 prevented AKT inactivation in response to AO treatment. Moreover, inhibiting USP30 decreased AKT levels in Hela Parkin USP30 cells and Jurkat T leukemia cells during mitochondrial stress and chemotherapies, theraby inducing cell apoptosis. Furthermore, We performed a chemical screening, suggesting that USP30 inhibitors may synergize with AKT/mTOR inhibitors in treating leukemia. Taken together, we demonstrated that Parkin and USP30 might regulate the AKT/mTOR signaling and cell survival during mitophagy, suggesting USP30 may serve as a potential drug target for leukemia treatment.

## Result

### Parkin and USP30 Regulate Mitophagy Independent Cell Apoptosis in Response to Mitochondrial Stress

**FIGURE 1:**
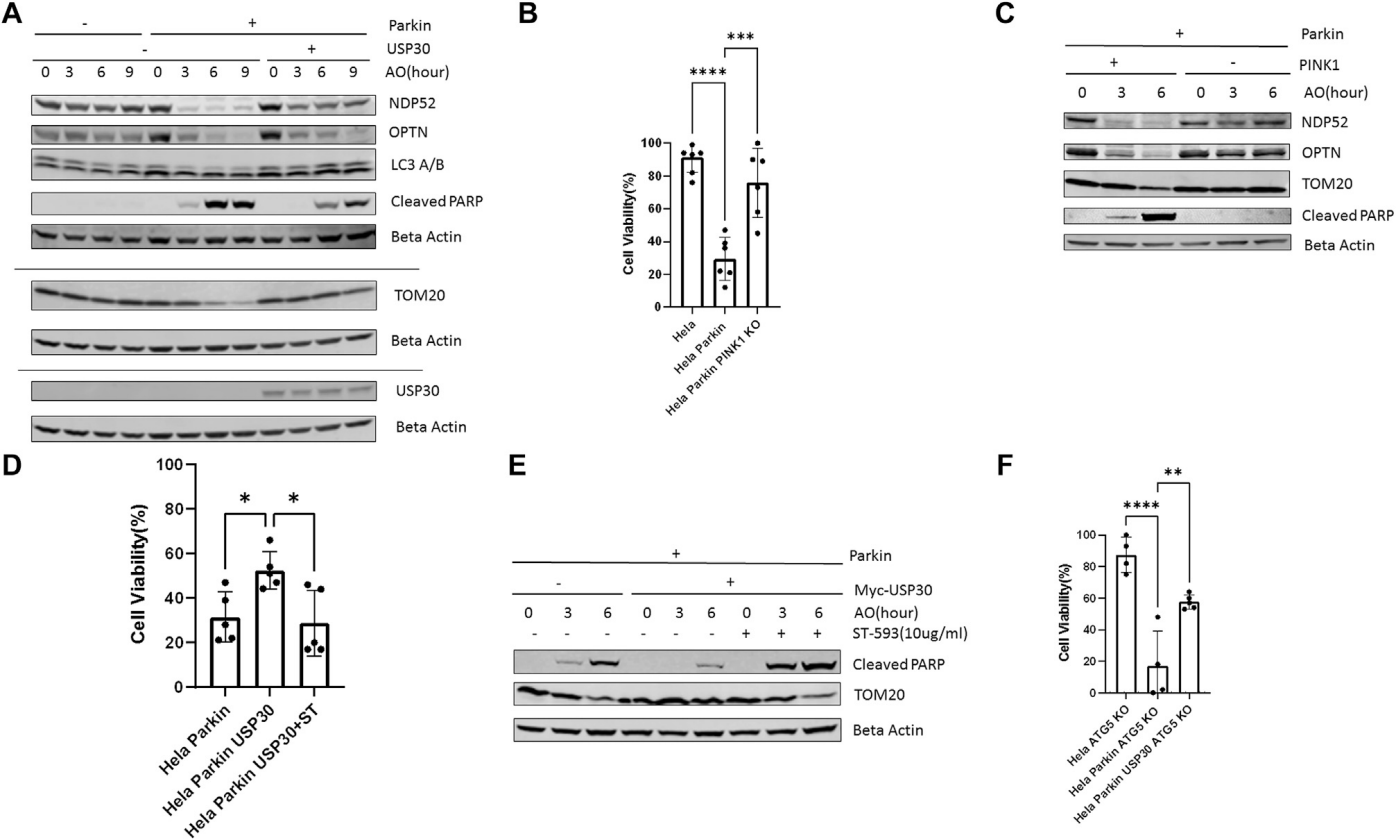
PINK1/Parkin-dependent mitophagy induces apoptosis during mitochondrial stress **(A)**. Western blot analysis of mitophagy proteins and the pro-apoptosis signal in Hela (no Parkin) cells and Hela Parkin cells after the AO (Antimycin A+ oligomycin) treatment. Cells were treated with AO at 5 ug/ml for 0, 3, 6, and 9 h. Beta Actin served as the loading control. These are representative figures from three independent experiments **(B)**. Cell viability assay of Hela (no Parkin) cells and Hela Parkin cells after AO treatment. Cells were treated with AO at 5 ug/ml for 24 h and then incubated with resazurin for 2 h. Fluorescence was read using 544 nm excitation and 590 nm emission wavelength. It is a representative figure from three independent experiments **(C)**. Western blot analysis of mitophagy proteins and the pro-apoptosis signal in Hela Parkin cells and Hela Parkin with PINK1 KO cells after the AO treatment. Cells were treated with AO at 5 ug/ml for 0, 3, 6, and 9 h. Beta Actin served as the loading control **(D)**. Cell viability assay of Hela Parkin cells and Hela Parkin with USP30 overexpression cells after AO treatment with or without ST-539. Cells were treated with AO at 5 ug/ml w/o 10 ug/ml ST-539 for 24 h and then incubated with resazurin for 2 h. Fluorescence was read using544 nm excitation and 590 nm emission wavelength **(E)**. Western blot analysis of mitophagy proteins and the pro-apoptosis signal in Hela Parkin cells and Hela Parkin with USP30 overexpression cells after the AO treatment w/o ST-539. Cells were treated with AO at 5 ug/ml w/o 10 ug/ml ST-539 for 0, 3, and 6 h. Beta Actin served as the loading control **(F)**. Cell viability assay of Hela ATG5 knockout cells, Hela ATG5 knockout Parkin cells, and Hela ATG5 knockout Parkin USP30 cells after AO treatment. Cells were treated with AO at 5 ug/ml for 24 h and then incubated with resazurin for 2 h. Fluorescence was read using544 nm excitation and 590 nm emission wavelength.

We treated Hela Parkin cells and Hela Parkin USP30 cells with a cocktail of the mitochondrial complex III inhibitor Antimycin A and the ATP synthase inhibitor Oligomycin (AO) for up to 9 h (Baudot et al., 2015; Zachari et al., 2019). NDP52 and OPTN are adaptor proteins that link ubiquitinated mitochondria to the autophagosome. These two proteins are degraded along with the mitochondria during mitophagy (Lazarou et al., 2015; Zachari et al., 2019). As expected, Parkin induced rapid mitophagy during AO treatment, shown as the degradation of TOM20, NDP52, and OPTN in Figure 1, a. PARP is a universal protein cleaved only during apoptosis (Gobeil et al., 2001; D'Amours et al., 2001). In the presence of Parkin, AO treatment resulted in an increase of cleaved PARP, suggesting an increase in cell apoptosis (Figure 1A). Consistent with the western blot results, our cell viability tests (by resazurin assay) indicated substantial cell death after 24 h of AO treatment (Figure 1B). The decrease in AKT and cell apoptosis required Parkin to be activated because knocking out PINK1, Parkin’activator, abolished mitophagy and cell apoptosis (Figures 1B,C). In Hela Parkin USP30 cells, we observed increased USP30 protein levels compared to Hela and Hela Parkin cells (Figure 1A and Supplementary Figure 1A). In addition, USP30 overexpression prevented mitophagy and reduced cell apoptosis following AO treatment (Figures 1A,D). By treating cells with 10 ug/ml ST-539, a specific USP30 inhibitor (Luo et al., 2021), Parkin mediated mitophagy, and cell apoptosis resumed (Figures 1D,E). We next asked whether the Parkin/USP30-mediated cell apoptosis during mitochondrial stress is mitophagy dependent. Autophagy-Related-Gene 5 (ATG5) is essential for autophagosome formation. Knocking out ATG5 undermines autophagy and mitophagy (Mai et al., 2012; Ye et al., 2018; Zheng et al., 2019). Cell viability data showed that knocking out ATG5 did not limit cell death during mitochondrial stress (Figure 1F), suggesting the Parkin/USP30-regulated cell apoptosis during mitochondrial stress is mitophagy independent. To summarize, Parkin promotes apoptosis, while USP30 antagonizes mitophagy-independent cell apoptosis during mitochondrial stress.

### Parkin and USP30 Regulate AKT/mTOR Signaling and Cell Survival in Response to Mitochondrial Stress

**FIGURE 2:**
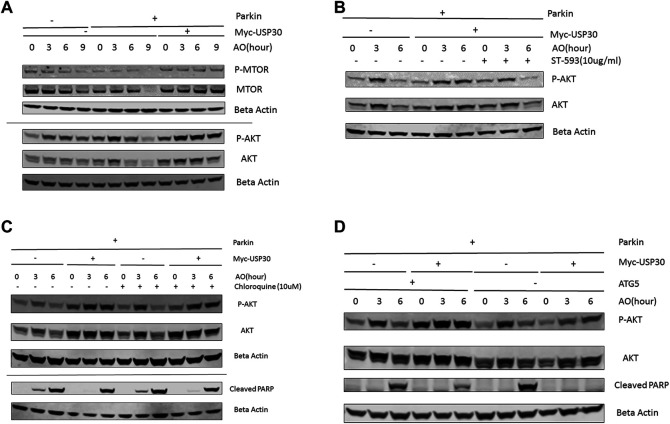
USP30 upregulates AKT/mTOR signal **(A)**. Western blot analysis of AKT/mTOR pathway proteins in Hela Parkin cells and Hela Parkin with USP30 overexpression cells after the AO treatment. Cells were treated with AO at 5 ug/ml for 0, 3, 6, and 9 h. Beta Actin served as the loading control. 2 **(B)**. Western blot analysis of AKT signal in Hela Parkin cells and Hela Parkin with USP30 overexpression cells after the AO treatment w/o ST-539. Cells were treated with AO at 5 ug/ml w/o ST-539 for 0, 3, and 6 h. Beta Actin served as the loading control. 2 **(C)**. Western blot analysis of AKT and cleaved PARP in Hela Parkin and Hela Parkin USP30 cells after the AO treatment w/o chloroquine. Cells were treated with AO at 5 ug/ml for 0, 3, and 6 h with DMSO or 10 uM chloroquine to inhibit autophagy/mitophagy. Beta Actin served as the loading control. 2 **(D)**. Western blot analysis of AKT and cleaved PARP in Hela ATG5 knockout Parkin and Hela ATG5 knockout Parkin USP30 cells after the AO treatment. Cells were treated with AO at 5 ug/ml for 0, 3, and 6 h. Beta Actin served as the loading control.

AKT/mTOR signal functions as a master signal for biogenesis and cell survival (Xu et al., 2020; Manning and Toker, 2017). Previous studies have indicated that the AKT/mTOR signal responds to multiple cellular stressors to promote cell survival (de la Cruz López et al., 2019; Xu et al., 2020; Yang et al., 2017). To investigate how the AKT/mTOR signaling responds to mitochondrial stress, we analyzed the protein levels of AKT, mTOR, P70S6K, and 4EBP1 in three cell lines: wild-type Hela cells, Hela Parkin cells, and Hela Parkin USP30 cells, following AO treatment. Western blot results indicated that the AKT/mTOR signaling increased throughout AO treatment, suggesting that AKT is activated and upregulated in response to mitochondrial stress (Figure 2, a), consistent with previous studies that indicate that mitochondrial stress activates the AKT survival pathway (Yang et al., 2017; Guha et al., 2010). In Hela Parkin cells, Akt and mTOR protein levels decreased significantly after 6 h of AO treatment (Figure 2A and Supplementary Figures 2A,B). The downstream signals of the AKT/mTOR pathway, 4EBP1 and P70S6K, were also downregulated (Supplementary Material 2C–E). These result suggest that Parkin may suppress AKT/mTOR signaling during mitophagy. USP30 overexpression significantly increased AKT’s protein level and upregulated AKT/mTOR signals during mitophagy (Figure 2A and Supplementary Figures 2A–E). Moreover, the addition of ST-539, a USP30 inhibitor, resulted in decreased AKT protein levels during mitophagy (Figure 2B). To test if mitophagy activity contributes to the regulation of AKT protein levels and cell apoptosis, we utilized Chloroquine, a lysosomal inhibitor, to inhibit mitophagy (Redmann et al., 2017; Mauthe et al., 2018). We demonstraed that the AKT protein levels and cleaved PARP following AO treatment were not affected after the addiotion of Chloroquine (Figure 2C). Similar results were observed in Hela Parkin and Hela Parkin USP30 cells that lack ATG5. (Figure 2D). These results suggest that AKT/mTOR signaling is activated in response to mitochondrial stress, and can be regulated by Parkin and USP30.

### USP30 May Serve as a Therapeutic Target for Leukemia Treatment

**FIGURE 3:**
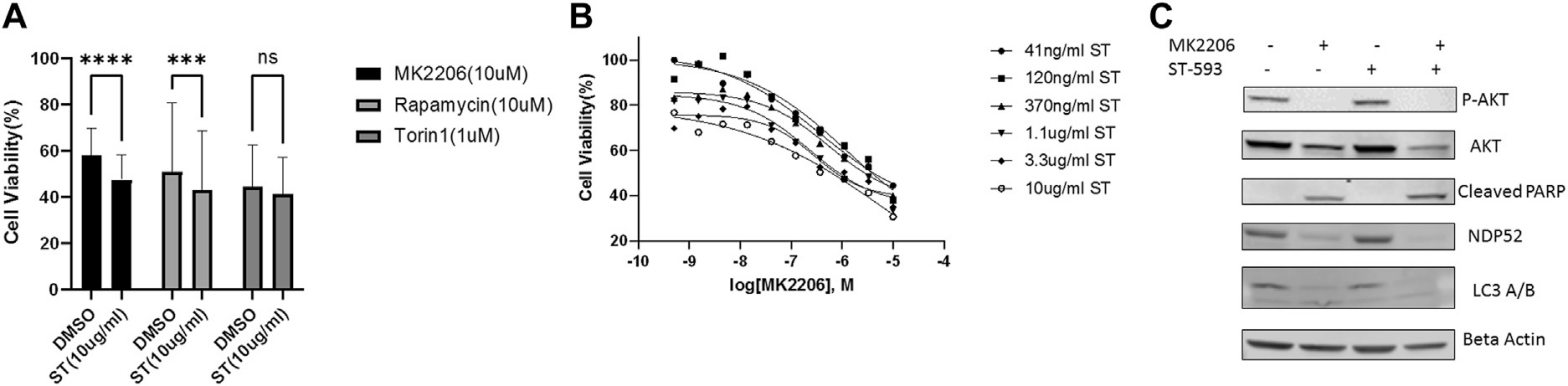
USP30 in cancer treatment. 3 **(A)**. Cell viability assay of Hela Parkin USP30 cells after AKT/mTOR inhibitors treatment w/o ST. Cells were treated with 10 uM MK2206 or 10uM Rapamycin or 1uM Torin1 for 48 h with DMSO or 10 ug/ml ST-539 and then incubated with resazurin for 2 h. Fluorescence was read using 544 nm excitation and 590 nm emission wavelength. 3 **(B)**. Cell viability assay on Jurkat T cells after 72 h MK2206 treatment with ST. Cells were treated with MK2206 and ST in the concentration gradient manner for 72 h. After the treatment, cells were incubated with resazurin for 2 h. Fluorescence was read using544 nm excitation and 590 nm emission wavelength. Each dot is the mean value of three biologically independent experiments. Trend lines are non-linear regression fitting curves. 3 **(C)**. Western blot analysis of AKT, mitophagy, and pro-apoptosis signal in Jurkat T cells treated with DMSO, MK2206, or ST. Cells were treated with DMSO, MK2206, ST, or MK220 + ST for 24 h. Beta Actin served as the loading control.

We next asked whether USP30 could be a viable target to synergize with AKT/mTOR inhibitors for leukemia treatment. We analyzed USP30’s effect on AKT/mTOR inhibitors by measuring the viability of Hela Parkin USP30 cells after treatment with MK2206 (10uM), Rapamycin (10uM), or Tronil1 (10 nM) in the presence and absence of ST-539. The cell viability analysis showed that inhibiting USP30 promoted drug-induced apoptosis significantly (Figure 3A). The apoptosis in treated cells suggests that USP30 inhibitors combined with AKT/mTOR inhibitors might prove a benefit in treating leukemia. The next set of experiments focused on Jurkat cells. Jurkat cells are immortalized human T lymphocytes used to study acute T cell leukemia (Abraham and Weiss, 2004). We evaluated Jurkat cells viability after MK2206 and ST-539 treatment. Jurkat cell growth data revealed that the addition of ST-539 significantly improved MK2206s efficacy in inhibiting cell growth. (Figure 3B). Western blot results indicated that ST-539 synergized with MK2006 to inhibit AKT activity and increase pro-apoptotic signaling (cleaved PARP) in Jurkat cells (Figure 3C).

**FIGURE 4:**
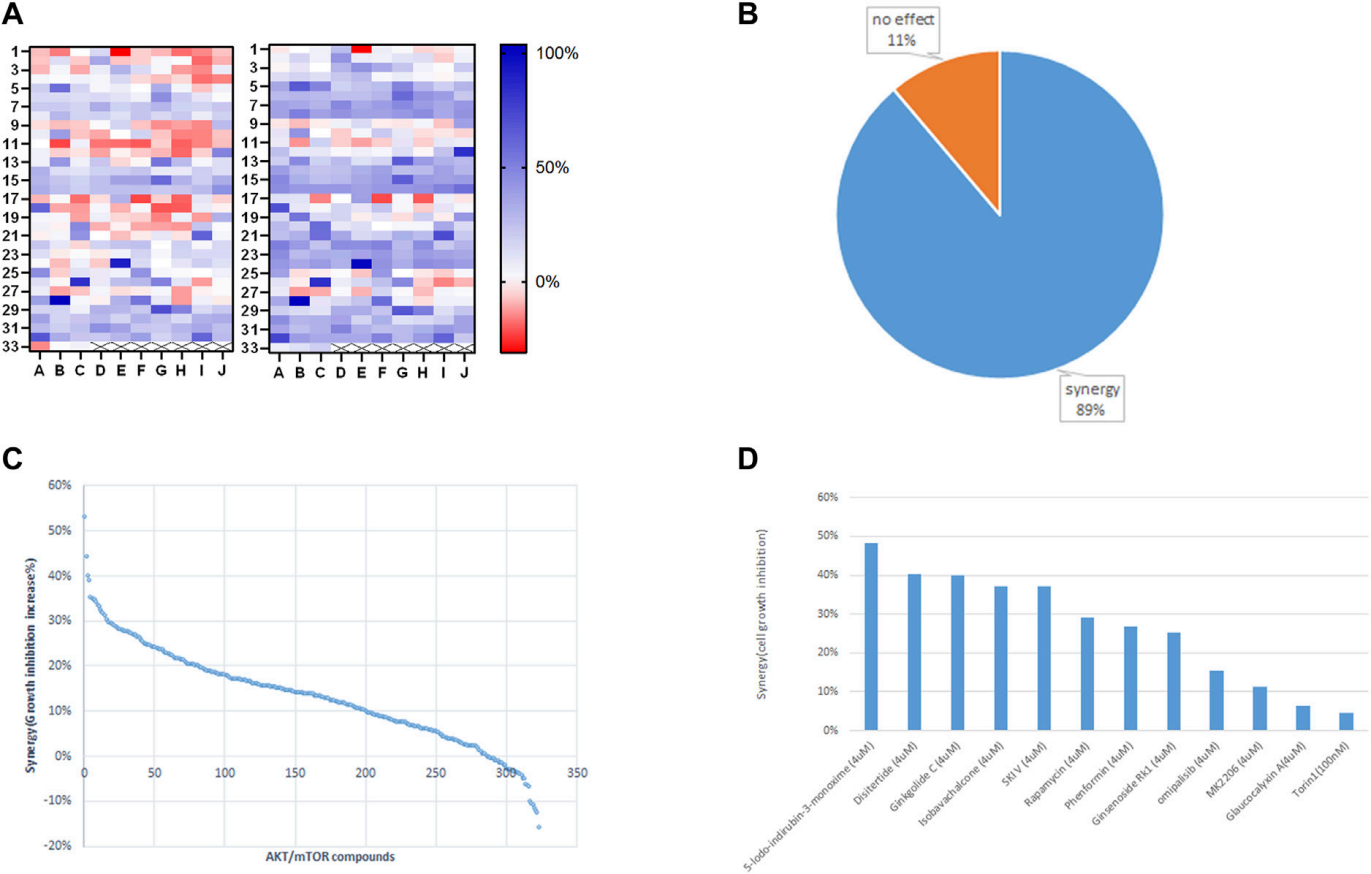
Small targeted chemical screen using the PI3K/Akt/mTOR compound library. 4 **(A)**. The heat map analysis of synergy between ST-539 and AKT/mTOR inhibitors on Jurkat cells growth inhibition. Jurkat T cells were treated with the compounds (4 uM) from PI3K/Akt/mTOR compound library (322 in total) w/o 10ug/ml ST-539 for 48 h, and the cell growth was analyzed by resazurin assay. The left one is the inhibition rate without ST-539, and the right one is the inhibition rate with ST-539. Data are the mean value from three biologically independent experiments. 4 **(B)**. The percentage of the compounds from PI3K/Akt/mTOR compound library synergize with ST-539 on Jurkat cells growth inhibition. 4 **(C)**. Ranks of synergistic effects between AKT/mTOR compounds and ST-539. Synergy is calculated by subtracting the growth inhibition caused by the compound alone from the growth inhibition caused by the compound and ST-539 together. 4 **(D)**. The synergy between specific compounds with ST-539. Data are the mean values from three biologically independent experiments.

**FIGURE 5:**
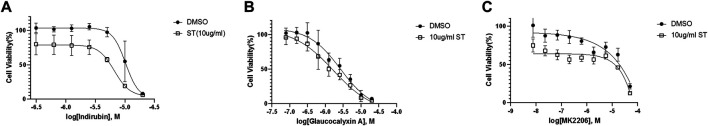
Selected inhibitors that show synergistic effects with ST-539 **(A)**. The dose-response curve of 5-lodo-indirubin-3-monoxime on Jurkat T cells w/o ST-539. Jurkat T cells were treated with 5-lodo-indirubin-3-monoxime in a concentration gradient manner with DMSO or 10 ug/ml ST-539 for 48 h. The cell growth was analyzed using a resazurin assay **(B)**. The dose-response of Glaucocalyxin A on Jurkat T cells w/o ST-539. Jurkat T cells were treated with Glaucocalyxin A in a concentration gradient manner with DMSO or 10 ug/ml ST-539 for 48 h. The cell growth was analyzed using a resazurin assay **(C)**. The dose-response of MK2206 on Jurkat T cells w/o ST-539. Jurkat T cells were treated with MK2206 in a concentration gradient manner with DMSO or 10 ug/ml ST-539 for 48 h. The cell growth was analyzed using a resazurin assay.

We employed Jurkat cells to investigate the synergistic effects between ST-539 and AKT inhibitors. We conducted a small-scale pilot chemical screen for Jurkat cell viability focusing on the AKT/mTOR compound library composed of 322 compounds, in the presence and absence of ST-539. Interestingly, 89% of the compounds work in synergy with ST-539 to significantly suppress cell proliferation (Figures 4A,B). These results suggest a synergistic effect between ST-539 and AKT inhibitors. Combined treatment of these inhibitors provides a unique approach to treat T cell leukemia. We ranked and reevaluated the most synergetic combinations from the previous experiment to find the most efficacious combinations (Figures 4C,D). The compound, 5-lodo-indirubin-3-monoxime (Indirubin), worked best with ST-539, suppressing cell growth by 48% (Figure 4D). The dose-response curve of indirubin, Glaucocalyxin A, and MK2206 w/o ST-539 showed that ST-539 promoted efficacy (Figures 5A–C). In short, combining USP30 inhibitors with AKT/mTOR compounds in treating leukemia warrants further investigation.

## Discussion

Whether mitophagy promotes or works as an agonist to cancer development is unclear. Mitophagy is vital in rewiring metabolic pathways to support cancer cells’ high energy demands (Chourasia et al., 2015; Vara-Perez et al., 2019). Previous studies induced cell apoptosis by knocking down USP30 potentiated BH3, ETC inhibitors (e.g., antimycin A, oligomycin), uncouplers (e.g., FCCP), subsequently boosting Parkin-dependent mitophagy. The increase in Parkin-dependent mitophagy indicates that USP30 could be a drug target in cancer treatment (Carroll et al., 2014; Liang et al., 2015). We have demonstrated that inhibiting USP30 boosts mitophagy and downregulates AKT signaling, promoting apoptosis during mitochondrial stress. Further drug screening using Jurkat cells demonstrates that the combination of USP30 inhibitors and AKT inhibitors is efficacious in treating T cell leukemia. However, whether Parkin and USP30 regulate AKT protein levels via modulating ubiquitination during mitochondrial stress remains to be investigated.

Overall, this research revealed the connections between Parkin, USP30, and AKT signals, proving that USP30 inhibitors may be effectivein leukemia treatment. Future studies could focus on investigating whether USP30 promotes AKT signaling and drug resistance in clinical status and whether this combined therapy works on patient-derived cancer cells and murine models.

## Methods

### Cell Culture and Reagents

All HeLa cell lines (wild-type, HeLa Parkin, HeLa Parkin PINK1 KO, HeLa Parkin USP30, HeLa Parkin Myr-AKT, HeLa Parkin Myr-AKT K179M, HeLa Parkin AKT-T308A/S473A, HeLa Parkin AKT-T308D/473D) were grown in Dulbecco’s minimum essential medium (DMEM) with 10% fetal bovine serum (FBS) supplemented and 1% penicillin-streptomycin. HeLa Parkin and HeLa Parkin PINK1 KO cells were previously described (Lazarou et al., 2015). Hela Parkin USP30 was generated using lentiviral vectors of pLVX-Puro-Myc-USP30, obtained from Addgene (Sowa et al., 2009). Jurkat T cells were grown in Roswell Park Memorial Institute Medium (RPMI-1640) with 10% fetal bovine serum (FBS) supplemented and 1% penicillin-streptomycin. For AO treatment, cells were incubated in a growth medium with 5 µM oligomycin and 5 µM antimycin A (details in figures). ST51000539 (ST-539) purchased from TimTec. Other chemicals were from Sigma-Aldrich (St. Louis).

### Western Blotting

Cells were lysed in RIPA buffer (50 mM Tris-HCl, at pH 8.0; 150 mM NaCl; 1% (vol/vol) Nonidet P-40; 0.5% sodium deoxycholate, 0.1% SDS and protease inhibitor cocktail (Roche)) on ice. Primary antibodies used as described: USP30 (Sino Biological Inc., 14,548-RP01, 1:500); *p*-AKT (Cell Signaling Technology, 4060S, 1:1000); Parkin (Cell Signaling Technology, 4211S, 1:1000); AKT (Cell Signaling Technology, 4685S, 1:1000); Cleaved PARP (Cell Signaling Technology, 5625S, 1:1000); OPTN (Proteintech, 10837-I-AP, 1:1000); *p*-mTOR (Cell Signaling Technology, 5536S, 1:1000); mTOR (Cell Signaling Technology, 2983S, 1:1000); TOM20 (Cell Signaling Technology, 42406S, 1:1000); NDP52 (Cell Signaling Technology, 60732S, 1:1000); p-P70S6K (Cell Signaling Technology, 9234P, 1:1000); P70S6K (Cell Signaling Technology, 9202S, 1:1000); LC3A/B (Cell Signaling Technology, 4108S, 1:1000); Beta-Actin (Cell Signaling Technology, 3700S, 1:1000). Secondary antibodies anti-rabbit (LI-COR, 926–32,211, 1:15 000) and anti-mouse (LI-COR, 926–68,072, 1:15 000) IgG were used to incubate membranes at room temperature for 1 h. Images were captured using the Odyssey system (LI-Cor).

### Drug Screening

Jurkat T cells were grown in 96-wells-plates with compounds from the PI3K/Akt/mTOR compound library bought from MedChemEXpress. Cells were treated with compounds at concentrations described in the figure for 48 h. The growth inhibition on cells was analyzed using a resazurin cell viability assay.

### Cell Viability Assay

Resazurin was bought from R&D Systems (AR002). Resazurin was added at a volume equal to 10% of the cell culture volume, and cells were incubated for 1 to 2 h at 37°. Fluorescence of the cell culture medium was read using 544 nm excitation and 590 nm emission wavelength.

## Data Availability

The original contributions presented in the study are included in the article/Supplementary Material further inquiries can be directed to the corresponding author.
