# Supplementary figures and images for: The Mitochondrial Deubiquitinase USP30 Regulates AKT/mTOR Signaling
Source: Front Pharmacol. 2022 Feb 17;13:816551. doi: 10.3389/fphar.2022.816551 (PMC8891576; doi:10.3389/fphar.2022.816551)

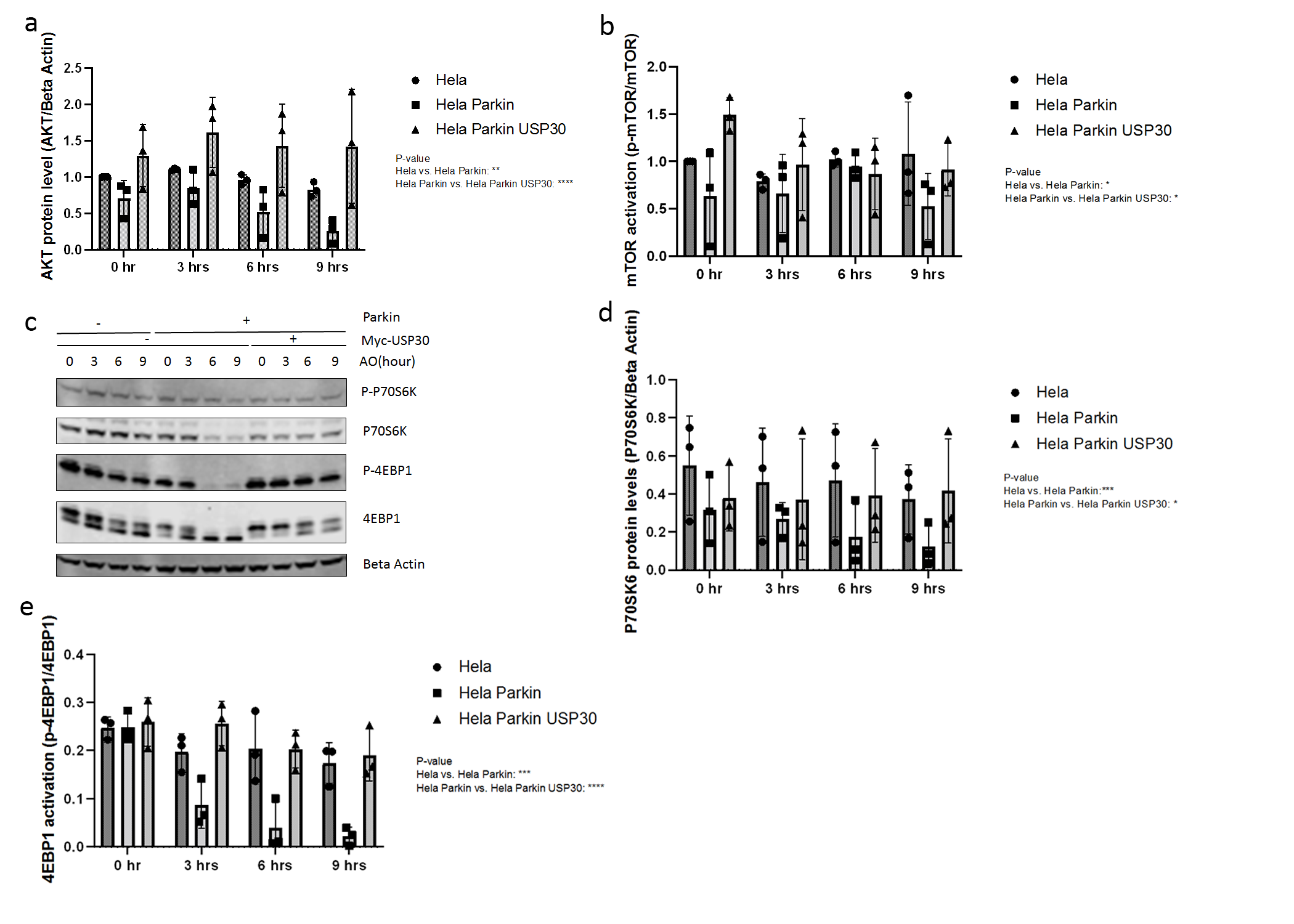

Supplement: Supplementary file 1 [file Image2.TIF]

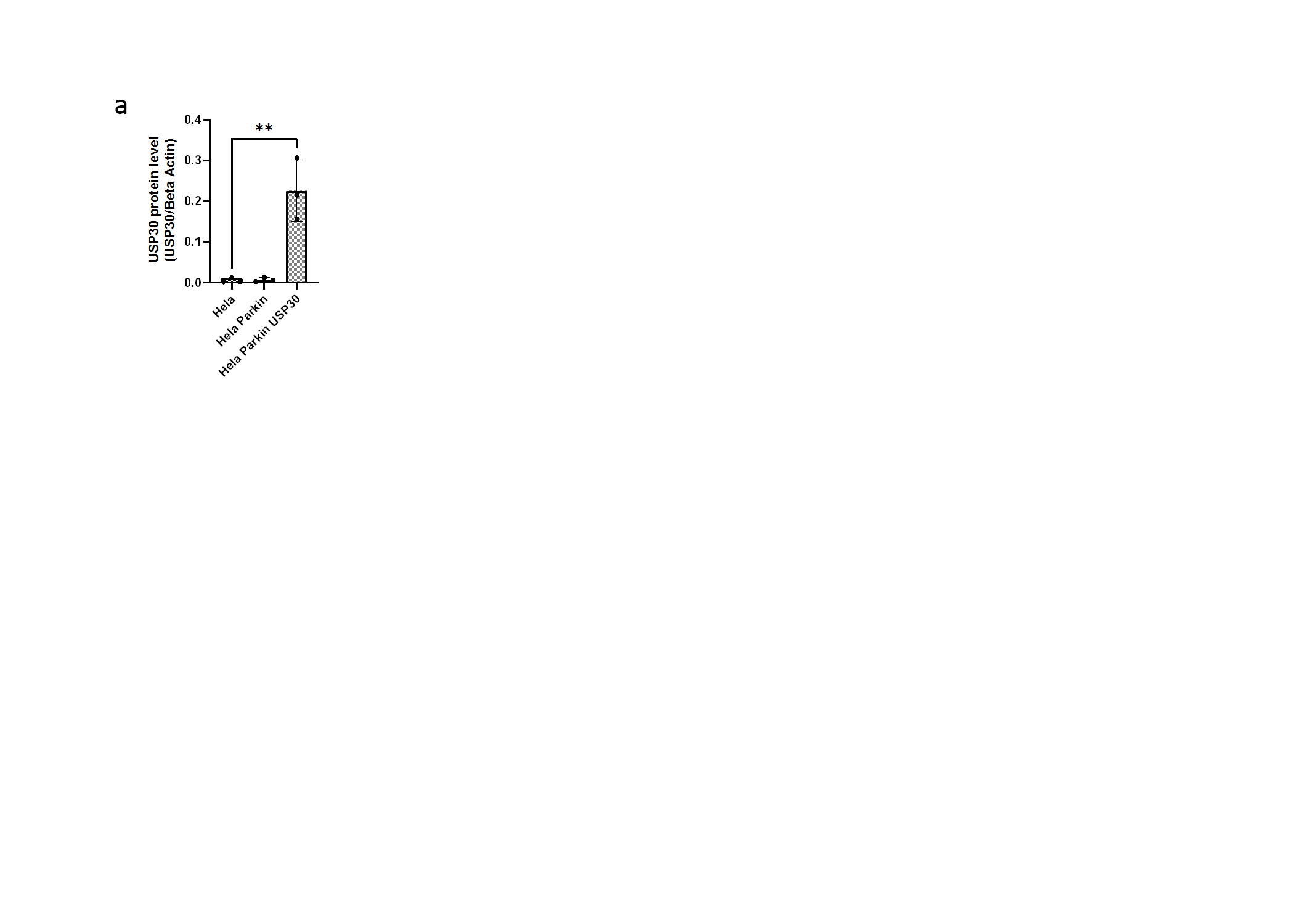

Supplement: Supplementary file 2 [file Image1.TIF]
